# Advances in Biomedical Functions of Natural Whitening Substances in the Treatment of Skin Pigmentation Diseases

**DOI:** 10.3390/pharmaceutics14112308

**Published:** 2022-10-27

**Authors:** Fan Liu, Linkai Qu, Hua Li, Jiaxuan He, Lei Wang, Yimeng Fang, Xiaoqing Yan, Qinsi Yang, Bo Peng, Wei Wu, Libo Jin, Da Sun

**Affiliations:** 1 Institute of Life Sciences & Biomedical Collaborative Innovation Center of Zhejiang Province, Wenzhou University, Wenzhou 325035, China; 2 College of Life Sciences, Jilin Agricultural University, Changchun 130118, China; 3 Key Laboratory for Biorheological Science and Technology of Ministry of Education, State and Local Joint Engineering Laboratory for Vascular Implants, Bioengineering College of Chongqing University, Chongqing 400044, China; 4 Chinese–American Research Institute for Diabetic Complications, School of Pharmaceutical Sciences, Wenzhou Medical University, Wenzhou 325000, China; 5 Wenzhou Institute, University of Chinese Academy of Sciences, Wenzhou 325000, China; 6 Wenzhou City and Kunlong Technology Co., Ltd. Joint Doctoral Innovation Station, Wenzhou Association for Science and Technology, Wenzhou 325000, China

**Keywords:** pigmentation diseases, natural whitening substances, melanin, melanogenesis, signaling pathway

## Abstract

Pigmentation diseases can lead to significant color differences between the affected part and the normal part, resulting in severe psychological and emotional distress among patients. The treatment of pigmentation diseases with good patient compliance is mainly in the form of topical drugs. However, conventional hydroquinone therapy contributes to several pathological conditions, such as erythema, dryness, and skin desquamation, and requires a longer treatment time to show significant results. To address these shortcomings, natural whitening substances represented by kojic acid and arbutin have gradually become the candidate ingredients of traditional local preparations due to their excellent biological safety. This review focuses on several natural whitening substances with potential therapeutic effects in pigmentation disease and their mechanisms, and a thorough discussion has been conducted into the solution methods for the challenges involved in the practical application of natural whitening substances.

## 1. Introduction

Skin pigments play a crucial role in protecting the skin from ultraviolet radiation (UVR) and oxidative stress from various pollutants [1]. However, abnormal pigmentation of the skin will lead to a variety of skin diseases, including melasma, pigmentation after inflammation freckles, and so on [2]. Pigmentation diseases have a significant impact on external appearance, leading to psychological and emotional distress and a reduction in the quality of life of affected patients, including men [3,4]. Although the vast majority of pigmentation disorders are benign or nonspecific, when evaluated and treated, patients often report feelings of shame, inferiority, anhedonia, dissatisfaction, lack of motivation to go outside, and, in serious cases, will suicidal tendencies [5].

Strict photoprotection is the main means to prevent pigmentation disorders, unfortunately, both the first-line treatment strategy of pigmentation disorder preparations and oral or emerging strategies (chemical skin peels and laser therapy) have certain limitations and side effects, such as erythema, skin desquamation, and dryness [6], and are accompanied by a large medical economic burden [7]. Therefore, due to low cost and ideal biocompatibility, natural whitening substances have the advantage of overcoming this problem [8]. In addition, in recent years, natural whitening substances have been favored by more and more consumers due to their mild, natural and healthy concept [8]. They are considered a substitute for chemically synthesized topical formulations for the treatment of pigmentation diseases and have become a hot spot in the field of cosmetics and medicine research [9].

Hence, this review focuses on the recent advances in biomedical functions of natural whitening substances in the treatment of skin pigmentation diseases and systematically summarizes the mechanism of natural whitening substances for applications in skin depigmentation diseases, especially highlighting the solution methods for the challenges involved in the practical application of natural whitening substances and anticipates their future perspective.

## 2. Mechanism in the Development and Progression of Pigmentation

**Figure 1:**
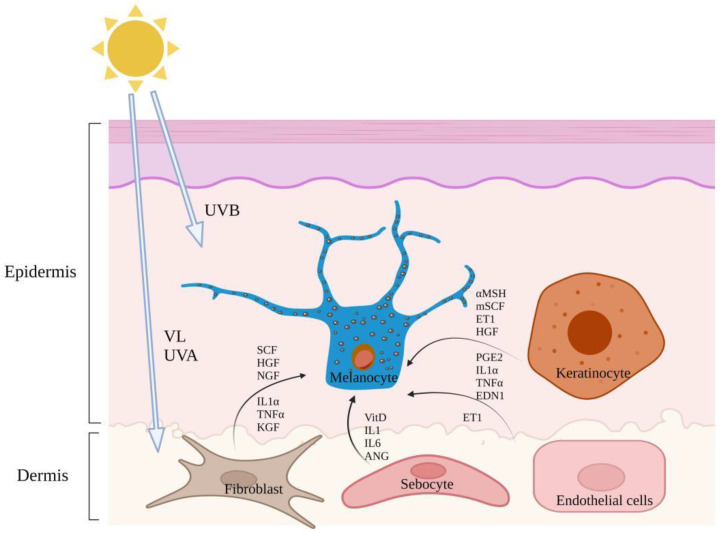
Melanin paracrine cytokines between skin cells interact with melanocytes. Increased melanin synthesis due to increased proliferation of melanocytes and increased tyrosinase (TYR) activity caused by paracrine secretion (Created with BioRender.com, accessed on 20 October 2022).

Skin pigmentation is a complicated biological process caused by various intrinsic and extrinsic factors. Extrinsic factors include ultraviolet (UV) exposure, drug effects, and dietary habits, while intrinsic factors are related to regulatory factors produced by cells involved in melanin production. UV solar radiation is important in the melanogenesis of melanocytes. Ultraviolet B (UVB) mainly damages the epidermis and superficial dermis, but ultraviolet A (UVA)/visible light (VL) can penetrate the basal layer and damage the dermis as a whole [10]. Melanin production is associated with the regulation of many paracrine factors. Particularly, as shown in Figure 1, paracrine melanogenic pathways are modulated by keratinocytes, fibroblasts, sebocytes, and endothelial cells secreting cytokines [11]. There are several important melanotropic paracrine cytokine networks between skin cells, including endothelin-1 (EDN-1), the membrane-bound stem cell factor (mSCF), proopiomelanocortin, prostaglandin E2 (PGE2), granulocyte-macrophage colony-stimulating factor, and basic fibroblast growth factor. Growth-regulated oncogene alpha and keratinocyte growth factor (KGF) are involved in keratinocyte/melanocyte interaction, soluble SCF, hepatocyte growth factor (HGF), and KGF are involved in fibroblast/melanocyte interaction [12]. Sebaceous glands can synthesize vitamin D (Vit D) and secrete different cytokines, such as interleukin-6, and growth factors, such as angiopoietin (ANG) and adipokines, which further directly or indirectly regulate melanocyte function [13]. Endothelial cells secrete endothelin-1 (ET-1) to induce the production of melanin. ET-1 can induce the angiogenic response of endothelial cells through the endothelin receptor B(EDNRB) and cooperate with the vascular endothelial growth factor to promote the formation of neovascularization in vivo [14].

**Figure 2:**
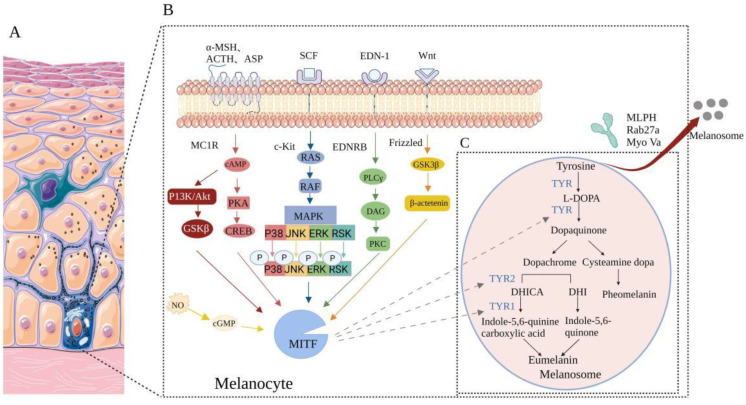
Schematic drawing of melanin synthesis, regulation, and transfer in melanocytes of skin epidermis and skin dullness. (**A**) Melanin produced by melanocytes is internalized by keratinocytes and then migrates and distributes to the surrounding membrane area, providing pigmentation. (**B**) Many cytokines activate signaling pathways by interacting with receptors on the melanin membrane and regulate the melanin production process through MITF. (**C**) Melanin production and transfer process (Created with BioRender.com, accessed on 20 October 2022).

As shown in Figure 2, melanin is synthesized and stored in melanosomes. Melanosomes are specialized in membrane-bound organelles in the melanocytes, influencing the ratio of eumelanin and pheomelanin through TYR activity and cysteine availability [15]. After melanosomes mature in melanocytes, they flow to the dendrite tip [16] and are further transported to the adjacent keratinocytes through pathways involving microtubule networks and actin cytoskeleton [17], whose dendrites can contact about 30–40 of keratinocytes [18]. Keratinocytes internalize the incoming melanosomes [19] and then migrate and distribute them to the peripheral membrane region, providing pigmentation and the darkening of skin color. A greater understanding of the regulatory pathway of melanin production may help identify more specific targets of drug action to effectively control pigmentation disorders. In melanocytes, the microphthalmia-associated transcription factor (MITF) is a basic-helix loop-helix leucine zipper transcription factor that belongs to the microphthalmia family [20]. Many cytokines activate signaling pathways by interacting with receptors on melanin membranes, including the phosphatidylinositol 3-kinase (PI3K)/Akt pathway, cyclic adenosine monophosphate (cAMP)/protein kinase A (PKA) pathway, mitogen-activated protein kinase (MAPK) pathway, EDN-1-mediated signaling cascade pathway and Wnt pathway [15,21]. The regulation of all signaling pathways involves MITF, which not only regulates the proliferation and survival of melanocytes but also regulates the expression of TYR, TYRP1, and TYRP2 [22]. Related proteins involved in melanosome transportation and distribution include melanophilin (MLPH), The ras-related protein (Rab27a) and myosin Va (Myo Va) [23] interact to form a terpolymer and participate in melanosome transport [24]. The regulatory pathways of melanin production are shown in Figure 2 MITF is activated by a variety of signaling pathways and binds to the m-box motif of the promoter region (TYR, TYRP-1, and TYRP-2 share a highly conserved sequence in the promoter region) and upregulates TYR, tyrosinase-related protein 1 (TYRP1) and tyrosinase-related protein 2 (TYRP2) to promote the synthesis of melanin [25]. TYR is a key enzyme that promotes melanin synthesis together with TYRP1 and TYRP2 [26]. The process of melanin production is first initiated by the hydroxylation of phenylalanine to L-tyrosine or directly by L-tyrosine, then hydroxylation to L-3, 4-dihydroxyphenylalanine (L-DOPA) and under the action of TYR, L-DOPA is further oxidized to an L-dopa quinone. Melanin can be divided into black-brown eumelanin and red-yellow pheomelanin. Without cysteine, the dopa quinone is converted into dopamine by intramolecular additions and decomposes spontaneously into 5, 6-dihydroxyindoles (DHI), forming eumelanin polymers [27] or by the transformation of TYRP2, the 5,6-dihydroxyindole-2-carboxylic acid (DHICA) is catalyzed by TYRP1 to generate indole-5, 6-quinone carboxylic acid, which eventually aggregates true melanin [28]. In the presence of cysteine, dopa quinone reacts with cysteine to produce cysteamine dopa, which is further converted into quinoline and finally polymerized to pheomelanin [29]. It is noteworthy that melanin produced in the basal layer does not cause visual darkening, and only when melanin is transferred to the cuticle can it cause visual darkening. Common conditions that may lead to pigmentation diseases are shown in Table 1.

## 3. Pigmentation Diseases

Skin depigmentation diseases are an umbrella term that includes any of the conditions associated with pigmentation, dullness, or the alteration of skin color. Common skin depigmentation diseases include melasma, post-inflammatory pigmentation, freckles, periorbital melanosis and so on. As a result of sun exposure, melasma is an acquired hypermelanosis that becomes worse with light exposure [40]. Post-inflammatory pigmentation (PIH), also known as acne pigmentation, is a pigmentation disorder caused by inflammation or damage to the skin, it can occur at any age and of any type and is a sequela of skin disease that afflicts many skin patients but is more common in individuals with darker skin [41]. Freckles are a common form of pigmentation and can usually be divided into ephelides and lentigines. They are benign spots of pigmentation that occur mainly in Caucasians and Asians with lighter skin [42]. Bastiaens et al. showed that lentigines were strongly positively correlated with age, while ephelides were negatively correlated with age [43]. Periorbital melanosis, commonly known as “dark circles under the eyes”, is a growing cosmetic problem that makes a patient’s exterior look prematurely old and can be painful [44]. More information is summarized in Table 2.

## 4. Biomedical Functions of Natural Whitening Substances on Pigmentation Diseases

**Figure 3:**
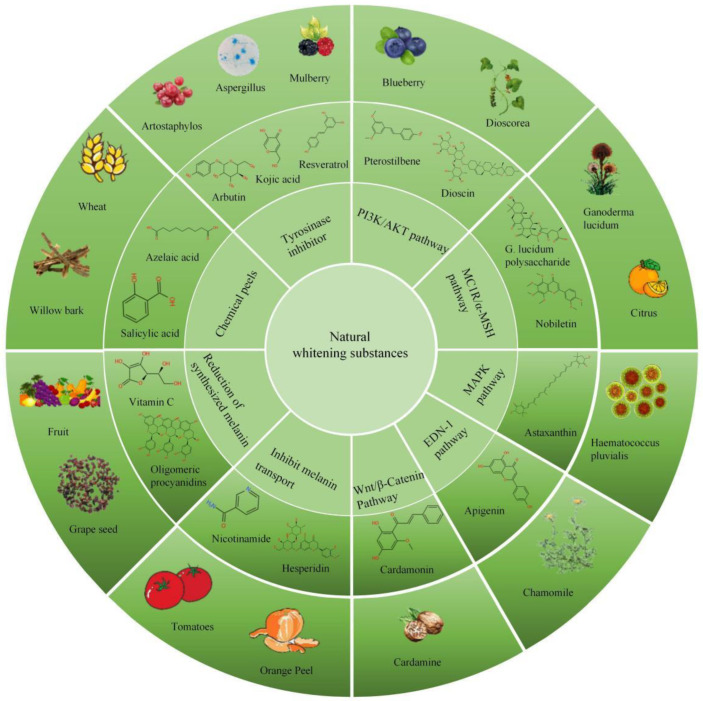
Natural whitening substances that act through different pathways.

Skin pigmentation can be improved by inhibiting melanin production, transportation, and deposition. Therefore, skin whitening can be achieved by inhibiting the activity of TYR, the key enzyme of melanin synthesis, blocking the cell signal transduction of the necessary melanin generation, inhibiting melanin transfer, and so on to prevent pigment deposition. The inhibition of TYR activity is a feasible method for both diseased and non-diseased skin. However, for skin lesions, blocking signaling in essential melanin-producing cells is a more ideal treatment for pigmentation. This article summarizes the various natural whitening substances from TYR inhibitors, melanin-producing intracellular signaling pathway blockers, melanin transport inhibitors, melanin-reducing agents, and chemical peeling agents. The classification and chemical structure of the related substances are shown in Figure 3.

### 4.1. Affect the Enzymes Needed in the Process of Melanin Production

Enzymes are one of the key influencing factors in all biochemical reactions in the body, and the prevention of melanin production and effective reduction in the amount of melanin can be achieved by inhibiting the enzymes necessary for each link in the process of melanin production in cells [62]. A copper-containing monooxygenase, TYR is a rate-limiting melanogenic enzyme involved in melanogenesis or melanin synthesis [63]. The TYR catalyzes two different reactions involving molecular oxygen in the melanin synthesis pathway from tyrosine: as a monophenolase when tyrosine is hydroxylated to L-DOPA and as a diphenolase when L-DOPA is oxidized to DOPA quinone [64]. However, few natural substances have been found to specifically act on monophenolase and diphenolase to inhibit melanin production, so this will be one of the potential targets to be explored in future research. Most whitening substances target TYR in the first two steps of the melanin synthesis reaction and inhibit its activity to achieve a whitening effect [65].

Bearberries of the *Ericaceae* and *Saxifragaceae* families of plants contain arbutin (also called beta-arbutin), which is a natural product [66]. Despite the fact that arbutin is composed of hydroquinone and D-glucose, it has been over 30 years since it has been studied for its skin-lightening effects [67]. Arbutin is a hydrophilic polyphenol that contains α-arbutin and β-arbutin in two configurations. The α-arbutin is usually produced by microorganisms or microbial glycosyltransferases. Studies have shown that α-arbutin has a stronger inhibitory effect on TYR than β-arbutin [68], where α-arbutin was 10 times more effective than β-arbutin [69]. It was found that the two arbutin’s had similar stability, both were stable in water and in methanol solutions in the absence of buffering or a stabilizer, both compounds were not stable under strong hydrolysis and enzyme conditions, and the stability of the application in cosmetics depended on the formulation type and pH value [70].

Kojic acid (KA) is a natural metabolite produced by the aerobic fermentation of fungi such as *Aspergillus* and *Acetobacter* [71]. KA can inhibit the activity of TYR, and the activation of nuclear factor kappa-B(NF-κB) in human keratinocytes may be related to melanin production, while kojic acid has a potential inhibitory effect on NF-κB activity in human keratinocytes [72]. The inhibition of TYR activity by KA may capture copper ions that decrease TYR activity when the skin is exposed to UVR [73]. KA is an ideal substitute for hydroquinone and has been shown to be effective in treating pigmentation disorders such as melasma [74]. KA is less irritating and is a rare and safe single whitening agent that can inhibit multiple enzymes at the same time. However, long-term use of KA still has certain cytotoxic effects [75]. KA is unstable to light, heat, and metal ions, has storage instability and poor skin absorption, and its use in cosmetics is limited. People have developed a large number of kojic acid derivatives to improve their performance, such as kojic acid dipalmitate and a vitamin C kojic acid ester [76,77].

Resveratrol is a natural polyphenol stilbene compound found mainly in grapes, mulberry and several other plants [78]. In previous studies, this natural molecule has been linked to anti-inflammatory and anti-cancer effects [79]. In addition, resveratrol has antioxidant properties, so it can protect cells from oxidative damage associated with the effects of free radicals and ultraviolet radiation on the skin by reducing the expression of AP-1 and NF-κB factors and slowing down the skin photoaging process [80]. Resveratrol has a strong inhibitory effect on the synthesis of melanin through a variety of mechanisms, which is related to its reduction in MITF activity [81]. Liu et al. showed that its inhibitory effect on melanin synthesis was achieved by inhibiting the expression of melanin enzymes such as TYR and TRP-1 [82]. However, the application of resveratrol in cosmetics is limited due to its poor chemical stability and bioavailability. Resveratrol derivatives can improve bioavailability and anti-melanogenesis activities, so many resveratrol derivatives have been developed and may be candidates for better applications in skin whitening cosmetics. Resveratrol triacetate is a stability-enhancing precursor that can be incorporated into cosmetic formulations to whiten human skin without causing irritation [83].

### 4.2. Melanin Synthesis Signaling Pathway Inhibitors

#### 4.2.1. PI3K/Akt Signaling Pathway Inhibitors

The primary intracellular target of cAMP is PKA, but it also regulates melanin production through a PKA-independent mechanism. PI3K is a phosphatidylinositol kinase that phosphorylates at the third hydroxyl group of the inositol ring and possesses phosphatidylinositol kinase activity and serine/threonine kinase activity [84]. Intracellular cAMP controls MITF expression by inhibiting PI3K and stimulating glycogen synthase kinase 3β (GSK3β) activity [85]. Pterostilbene (Pt) is a natural polyphenol found in blueberries and several grapes that has been extensively studied for potential health benefits [86]. Pt is a kind of resveratrol analog and its resistance to the melanin effect is more effective than resveratrol. In α-MSH-stimulated B16F10 cells, Pt (10–30 μM) down-regulated the expression of TYR, TRP-1, TRP-2, MITF, and melanin and inhibited the PI3K/AKT pathway [87]. Diosgenin is composed of spirostane-type diosgenin and a sugar chain connected to the 3-hydroxyl group by glycosidic bonds. It mainly exists in Dioscorea, Liliaceae, Caryophyllaceae, Rosaceae, and other plants.

Studies have shown that diosgenin can regulate the decrease in PI3K expression and phosphorylated AKT and mTOR [88], which will affect the phosphorylation of downstream GSK3β and down-regulate the expression of MITF. MITF is the most important transcription factor in melanin synthesis, which will lead to a decrease in melanin synthesis.

#### 4.2.2. Melanin Production Is Inhibited by Inhibiting MC1R/α-MSH Signaling Pathway

MC1R/α-MSH signaling pathways, also known as the camp-dependent signaling pathway, are mainly regulated by the cAMP/PKA factor. Gene MC1R encodes a seven-transmembrane G protein-coupled receptor for MC1R on 16q24.3 [89]. MC1R signaling is a major determinant for the amount and type of melanin pigments synthesized by melanocytes, regulating both basal pigmentation and UV-induced tanning responses [90]. UV irradiation triggers the binding of MC1R by keratinocyte-derived α-MSH, resulting in the activation of cAMP signaling and the stimulation of DNA repair in melanocytes [91]. *Polysaccharides G. lucidum polysaccharide* (GLP) is the most abundant active ingredient in *Ganoderma lucidum*, which has been reported to have effects on skin whitening and skin cancer [92]. GLP effectively inhibits UVB-induced melanogenesis by inhibiting cAMP/PKA and ROS/MAPK signaling pathways [93]. In addition, GLP can inhibit the paracrine effect of keratinocytes and fibroblasts to reduce melanin production [94]. One of the promising compounds under extensive research is nobiletin, which is a polymethoxyflavone derived exclusively from citrus peels and has anti-inflammatory and anti-melanin production [95]. Nobiletin reduces MITF and cAMP/PKA-induced TYR activity and cAMP-response element binding protein (CREB) phosphorylation in a dose-dependent manner [96] and effectively reduces the ET plus SCF-induced phosphorylation of RAF-1, MEK, and extracellular-regulated protein kinases (ERK1/2).

#### 4.2.3. MAPK Pathway Inhibitors

The MAPK cascade is a conserved signaling module maintained in eukaryotes, where the phosphorylation of proteins regulates cell proliferation, differentiation, and death [97]. Mammals express at least four groups of significantly regulated MAPK, ERK1/2, Jun amino-terminal kinase 1/2/3, P38 protein, and ERK5 [98]. ERK or c-Jun NH2-terminal kinase (JNK) phosphorylation triggers MITF expression, resulting in degradation and the subsequent JNK downregulation of melanin production. In contrast to this pathway, the activation of ERK can lead to the phosphorylation of CREB, and the phosphorylated CREB binds to the common motifs of CREB in the MITF promoter region and is modulated to express the MITF gene. The phosphorylation of P38 activates MITF expression, which in turn up-regulates melanin-producing related proteins, thereby affecting melanin synthesis.

Several stress conditions can increase astaxanthin accumulation in *Haematococcus pluvialis*: a unicellular algae [99]. Astaxanthin is a ketone carotenoid that inhibits melanin production by disrupting SCF-induced intracellular signaling and eliminates the human epidermal equivalent (HEE) pigmentation stimulated by SCF. However, it was unable to interrupt the intracellular cascade triggered by endothelin (EDN) in HEE [100]. Quercetin-3-O-β-d-glucopyranosyl-(1→6)-β-d-glucopyranosid (QCGG) was isolated from the calyx of Persimmon (*Diospyros kaki*). QCGG is activated by ERK, which reduces the expression of tyrosine and tyrosine-associated proteins and subsequently down-regulates CREB, P38, and MITF, thereby reducing melanin synthesis [101].

#### 4.2.4. EDN-1 Mediated Signaling Cascade Inhibitors

The interaction of EDN with its receptor EDNRB is one of the key paracrine interactions between keratinocytes and melanocytes [102]. EDN-1 binds to EDNRB and triggers the hydrolysis of polyphosphatidylinositol, which produces inositol triphosphate and diacylglycerol through the action of activated phospholipase C γ, which aggregates intracellular Ca^2+^ and activates PKC, respectively. Activated PKC directly phosphorylates RAF or RAF-1, which leads to the activation of the MAPK cascade.

*Matricaria recutita* L. is an annual to perennial herbaceous plant in the chrysanthemum family. Chamomile extract pre-incubation can disrupt the EDN1 induction of calcium mobilization and the chamomile extract on the TYR activity in vitro inhibitory effect, while chamomile extracts immediately after UVB exposure in guinea pig skin topical application at 2 weeks, useing only 10% arbutin or only carrier therapy compared to UVB-induced pigmentation which experienced a significantly reduced strength [12].

#### 4.2.5. Wnt/β-Catenin Signaling Pathway Inhibitors

Evolutionarily conserved cysteine-rich, secreted glycoproteins, Wnts are central regulators of vertebrate and invertebrate development, affecting cell proliferation, differentiation, and migration [103]. Among the main branches of the Wnt signaling pathway is the Wnt/β-catenin pathway, in which Wnt proteins bind to Frizzled receptors and activate Dishevelled [104]. The β-catenin destruction complex is then inactivated, leading to the aggregation of unphosphorylated β-catenin in the cytoplasm and nucleus [105]. Wnt activation induces the translocation of β-catenin to the nucleus, where the β-catenin binds to TCF/LEF to induce the transcription of Wnt target genes [106]. Increased levels of nuclear β-catenin can increase the expression of MITF, thereby increasing the survival and proliferation of melanoma cells and stimulating melanin production [107].

Cardamonin is found mainly in the ginger plant *Alpinia katsumadai Hayata* and is a natural source of chalcan. Cardamonin promotes the degradation of β-catenin to inhibit the Wnt/β-catenin signaling and inhibits melanin production by down-regulating MITF and TYR expression, resulting in a reduced melanin content [108]. Argan Press Cake (APC) was extracted from the kernels of *Argania spinosa*. In the study of Thouria Bourhim et al., APC-inhibited MITF expression and down-regulated Wnt/β-catenin signaling pathways, and, in addition, APC also prevented melanosome transportation by down-regulating Rab27a [109].

### 4.3. Inhibit Melanin Transport Process

Melanin that has been produced cannot cause visual “blackening” if it is not transported smoothly to the cuticle. So if you can inhibit the transport of melanin so that it does not reach the surface of the skin, it does not cause darkening, which is another angle of whitening, where melanin is produced but is not visible to the naked eye [110].

Nicotinamide, also known as nicotinamide, is a B3 vitamin widely found in plants and animals [111]. Nicotinamide interferes with the interaction between keratinocytes and melanocytes by modulating protease-activated receptors involved in melanosome metastasis [112]. Nicotinamide is gaining increasing attention for its whitening properties, stimulating DNA repair, and ability to inhibit cancer caused by ultraviolet light [113]. Relevant studies show that nicotinamide reduces mast cell filtrate and improves solar elasticity in patients with melasma, with fewer side effects [114]. Hesperidin is a large number of flavonoids isolated from citrus, and it has certain therapeutic effects in various diseases and has anti-inflammatory and antioxidant properties [115]. Hesperidin inhibited the transport of melanin bodies in melanocytes by blocking the interaction between Rab27a and MLPH and showed a reduction in skin pigmentation in 3d-cultured human skin models [116]. In addition, relevant studies have shown that topical hesperidin can enhance skin barrier functions [117] and prevent the glucocorticoid-induced epidermal barrier dysfunction in mice [118]. By contrast, Usach et al. reported that hesperidin induces melanin production in human melanocytes by enhancing TYR activity in a dose-dependent manner [119]. Therefore, it is necessary to further evaluate and study the decolorization characteristics of hesperidin.

### 4.4. Reduction of Synthesized Melanin

The original agent can reduce the oxidation products in the process of melanin generation and block the generation of melanin through the continuous reduction in oxidation products. At the same time, the reducing agent can desalinate melanin and inhibit the production of dopamine, the oxidation product of dopaquinone. In addition, the reducing agent can also inhibit the free radical activity caused by ultraviolet light and haze so as to slow down the aging and dark skin color caused by the oxidation of the skin collagen [120]. Composition with such whitening mechanisms mainly includes vitamin C(VC) and its derivatives (oxidizing melanin can be colorless reducing melanin, and to a certain extent, inhibiting TYR activity, oligomeric procyanidins (dopamine quinone reductants, can restore melanin, inhibit the generation of fat brown pigments, age spots, and can also inhibit tyrosine enzyme activity).

VC, also known as ascorbic acid, is a water-soluble vitamin that humans and other primates cannot synthesize and must be obtained through food [121]. VC usually exists only in plant food, as animal food basically does not contain VC. Instead, all kinds of fresh vegetables and fruits are its main source. VC may have a good therapeutic effect on patients with melasma or post-inflammatory pigmentation. VC treatment of melanocytes resulted in a significant decrease in TYR activity, melanin content, and intracellular ROS levels, indicating that VC has anti-melanin and antioxidant activities [122]. VC is generally well tolerated and safe, even in large doses [123]. VC can protect cells from oxidative stress when ROS is reduced, but it is easily oxidized by the external environment, such as temperature, pH and air [124].

Oligomeric procyanidins (OPC) exist in the skins, shells, seeds, nuclei, flowers, and leaves of various plants. They are biological flavonoids with special molecular structures and are effective natural antioxidants in scavenging free radicals in the human body [125]. It can inhibit the activity of TYR. It can reduce the phthalic quinone structure of melanin to phenolic structures and make the pigments fade. It can inhibit the Maillard reaction due to the amino of protein and nucleic acids and inhibit the formation of lipofuscin and senile plaques. Studies have shown that the long-term use of OPC can significantly reduce the skin melanin index and enhance the water content of cuticles [126].

### 4.5. Accelerate Skin Metabolism and Cuticle Shedding

Even if the melanin that has been produced reaches the skin surface smoothly, there is another way to play a certain whitening effect, which is to speed up the skin metabolism and accelerate the cuticle. Melanin that has reached the surface of the skin is shed along with the cells in the cuticle, which speeds up melanin metabolism and improves skin color [127]. Chemical peels are widely used in cosmetics and dermatology as a common treatment. Chemical peeling results in the thinning of the cuticle, epidermal lysis, and the dispersion of melanin in the basal layer, thereby removing melanin from the epidermal melanin and keratinocytes. The aim is to improve the damaged skin by removing the damaged skin. A superficial peel is one of the most effective ways to reduce acne scars and repair aging skin and various skin problems. Medium-depth peels are suitable for freckles, actinic keratosis, and fine, static wrinkles [128]. The whitening component that has this kind of mechanism is the acid kind material, including fruit acid, salicylic acid, and so on, because the whitening component, such as additional kojic acid has a certain acidity which can accelerate the corneous layer to metabolize the effect of speed likewise. It should be noted that people with sensitive skin and thin cuticles should carefully choose this method to avoid excessive damage to the cuticle and more skin problems.

Salicylic acid is a plant hormone, first found in the nature of willow bark, white leaves, and sweet birch, belonging to β-hydroxy acid and phenolic compounds; it has anti-inflammatory, antibacterial, decolorization, and other properties [129]. SA is a fat-soluble drug that can fuse with the epidermal and sebaceous lipids in hair follicles and penetrate deep into the cuticle and pores without causing irritation to keratinocytes [130]. Salicylic acid ethanol solution is suitable for the treatment of melasma, post-inflammatory pigmentation, and acne, especially for dark-skinned people, as an excellent peeling agent. The study showed that after 30% salicylic acid treatment, the total lesions improved in 95% of patients with mild and moderate acne vulgaris, in 85% of inflammatory lesions, and no complications occurred at the site of the lesions reported by the patients [131]. Chemical peels are one of the effective components of the melasma treatment strategy. For example, Sarkar et al. compared glycolic acid (35%) versus salicylic-mandelic acid (20% salicylic/10% mandelic acid) versus the phytic combination peels efficacy and tolerance in patients with melasma, the final result being that salicylic mandelic peels were better tolerated [132].

Azelaic acid, a saturated dicarboxylic acid naturally occurring in wheat, rye, and barley, is well tolerated and safe for a range of skin disorders, and monotherapy or combination therapies that may be an effective first-line or alternative therapy for a variety of pigmentation disorders, including common inflammatory pigmentation and melasma [133]. Azelaic acid reduces the secretion of growth factors and metalloproteinases such as SCF and HGF [134]. In Karolina Chilicka’s et al. study, patients treated with Azelaic acid had less oily skin and performed well in peeling after six sessions [135].

## 5. Conclusions and Perspectives

Natural whitening substances can whiten, protect and even repair the skin, but there are some negative effects in specific cases, the most common being skin allergies [136]. Therefore, after adding natural whitening substances to cosmetics or drugs, there must be an ingredient reminder label so that patients can reasonably choose products that suit them. In addition, natural substances may have some disadvantages, such as poor stability and low bioavailability, which can be improved by structural modification combined with carriers and surfactants [137,138,139]. At the same time, when using whitening products, we should also pay attention to the prevention of pigmentation. In daily life, light protection is an important means to prevent skin darkening induced by ultraviolet rays. Physical sunscreen containing red (Fe_2_O_3_), yellow (FeO), black (FeO Fe_2_O_3_), and titanium dioxide can prevent pigmentation caused by VL/high energy visible light [140]. Therefore, the combination of natural whitening substances and the above physical sunscreen is a good strategy. At the same time, further research on natural whitening substances can explore their potential to combine with standard therapies so as to achieve more effective control of pigmentation.

In short, the development of effective and preventive skin whitening agents from natural sources has a higher safety than synthetic substances with relatively non-toxic and fewer side effects and is expected to become a new active whitening substance. With the pursuit of safer and more effective whitening substances, the supply and demand of natural whitening substances are increasing significantly. Therefore, more clinical studies are needed to design and develop new products based on natural whitening substances.

## Figures and Tables

**Table 1:** Risk factors for pigmentation and their action pathways.

Conditions That May Lead to Pigmentation Disorders	Major Risk Factors	Action Pathway	References
Sunlight exposure	UVR	Enhanced production of reactive oxygen species (ROS) in keratinocytes and melanocytes, resulting in DNA damage of keratinocytes and further activation of tumor suppressor protein p53.	[30,31,32]
Foods with high metal content	Copper, iron, zinc and other metals	Directly or indirectly increase the amount and activity of TYR and the quantity and activity of dopa quinone related to melanin production.	[33,34]
Some drugs	Chloroquine, minocycline, bleomycin	The affinity for melanin is particularly strong, which will accentuate skin pigmentation.	[35,36,37]
Part of the disease	Malnutrition, urticaria, dermatitis, acne	Oxidation and antioxidant imbalance in the human body; endocrine disorders; microecological imbalance; metabolic disorders; abnormal trace element content in the body.	[38,39]

**Table 2:** The susceptible group, clinical features, pathological features, associated causes and pathogenesis of common pigmentation diseases.

Etiology	Susceptible Group	Clinical Features	Pathologic Features	Associated Causes	Pathogenesis
Melasma	Most of them are women of childbearing age but also occurs in men [45].	Light to brown pigmented, symmetrical spots with irregular, serrated shapes commonly found on the forehead, cheeks, upper lips, and chin [40].	Melanin production and melanocyte increases in the epidermal and dermal skin layers [5] and there are more mast cells in the elastic zone of degenerative melasma [46] and vascular proliferation [14]. Damage to the basement membrane promotes the descent of melanocytes and melanin into the dermis, which is represented by the free melanin or melanocytes often observed in the dermis of skin melasma [46].	Pregnancy, intake or oral contraceptives and hormone drugs, exposure to sunlight (UVA and UVB wavebands), etc., have an obvious genetic predisposition [5].	Skin fibroblasts secrete several stimuli including dickkopf-1, SCF, HGF, (nerve growth factor) NGF, (tumor necrosis factor-α) TNF-α and (Interleukin 1α) IL-1α [47], sebaceous glands synthesize Vit D and secrete various cytokines, such as interleukin-6 and growth factors such as angiopoietin and adipokine, which further regulate melanocyte function directly or indirectly [13]. Mast cells induce vascular proliferation through various angiogenic factors and destroy the basal layer through trypsin and granzymes B [46]. Vascular endothelial cells secrete ET1 to induce melanin production [14], most paracrine regulation of melasma involves the Wnt pathway.
Post-inflammatory Pigmentation	All skin types, especially those with darker skin colors [41].	PIH of epidermis was tan, brown, or dark brown; the PIH within the dermis has a bluish-gray appearance [48].	Melanocyte proliferation, increased the number of melanocyte dendrites and TYR, etc [49]. Elanocyte phagocytes exist in the dermis [50].	PIH can be caused by skin inflammation, burns, trauma, acne, and other endogenous and external factors that can cause skin tissue and cell damage, including physical factors, chemical factors, biological factors, tissue necrosis, allergies, and foreign bodies [51].	A variety of endogenous and exogenous factors act on the migration, proliferation, and differentiation of melanocytes. TYR synthesis and activation, melanosome transfer to keratinocytes and other terminal processes through inflammatory mediators, inflammatory cytokines, melanotrophs, NO and other inflammatory regulatory factors, result in the PIH effect [52,53].
Freckles	The onset of Ephelides usually occurs around the age of five, mostly in women and, in some people, it tends to decrease in adulthood [53].	Yellow brown or brown spots, symmetrical distribution on the face, neck, back, shoulder; mostly 1–2 mm in diameter with small macules and a smooth, clear edge.	Epidermal pigmentation increased but the quantity of melanocytes did not.	Genetic factors mostly: it is an autosomal dominant-inherited disease [53].	The (melanocortin 1 receptor) MC1R gene is the main gene for the formation of ephelides [43]. MC1R binds to the α-melanocyte-stimulating hormone(α-MSH) and adrenocorticotropic hormone (ACTH) to activate the cAMP pathway, which leads to melanin synthesis. IRF4 cooperates with MITF to activate the expression of TYR and affect the synthesis of melanin [54].
	Lentigines tend to be around 50 years.	Color varies from light yellow to dark brown to black; mainly distributed in the forehead, cheek, back of hand and forearm; they are round or oval and irregular in shape [55].	Epidermal melanocytes proliferate and dendrites increase [42].	Environmental factors, such as sunlight and air pollution, contribute to the development of lentigines [56].	IL-1α and TNFα are released from keratinocytes by autocrine and promote the up-regulation of EDN-1 and mSCF [57]. Fibroblast release of KGF acts directly or indirectly through keratinocytes, leading to pigmentation [58].
Periorbital melanosis	It can occur in both sexes and is more common in dark-skinned individuals.	Dark brown pigmentation may occur on upper and lower eyelids.	Dermal melanocytes increase [59] and blood vessels dilate.	Ocular melanin pigmentation, flabby skin, atopic dermatitis secondary inflammatory pigmentation and genetics.	Increase in dermal melanocytes, excessive pigmentation after inflammation, extension of facial pigment boundary [60], diffusion of superficial position of vascular system [61], edema around eyes when morning or salt intake is too much and lacrimal groove depression [61] may be caused.

## Data Availability

Not applicable.

## Abbreviations

UVR	Ultraviolet radiation
UV	Ultraviolet
UVB	Ultraviolet B
UVA	Ultraviolet A
VL	Visible light
EDN-1	Endothelin-1
mSCF	Membrane-bound stem cell factor
PGE2	Prostaglandin E2
KGF	Keratinocyte growth factor
HGF	Hepatocyte growth factor
VitD	Vitamin D
ANG	Angiopoietin
ET1	Endothelin 1
TYR	Tyrosinase
MITF	Microphthalmia-associated transcription factor
TYRP1	Tyrosinase- related protein 1
TYRP2	Tyrosinase- related protein 2
L-DOPA	L-3, 4-dihydroxyphenylalanine
DHI	5, 6-dihydroxyindoles
DHICA	5,6-dihydroxyindole-2-carboxylic acid
PI3K	Phosphatidylinositol 3-kinase
cAMP	Cyclic adenosine monophosphate
PKA	protein kinase A
MAPK	Mitogen-activated protein kinase
MLPH	Melanophilin
Rab27a	Ras-related protein
Myo Va	Myosin Va
ROS	Reactive oxygen species
PIH	Post-inflammatory pigmentation
NGF	Nerve growth factor
TNF-α	Tumor necrosis factor-α
IL-1α	Interleukin 1α
MC1R	Melanocortin 1 receptor
α-MSH	α-melanocyte-stimulating hormone
ACTH	Adrenocorticotropic hormone
KA	Kojic acid
NF-κB	Nuclear factor kappa-B
GSK3β	Glycogen synthase kinase 3β
Pt	Pterostilbene
GLP	G. lucidum polysaccharide
CREB	cAMP-response element binding protein
ERK1/2	Extracellular regulated protein kinases
JNK	c-Jun NH2-terminal kinase
EDN	Endothelin
HEE	Human epidermal equivalent
QCGG	Quercetin-3-O-β-d-glucopyranosyl-(1 → 6)-β-d-glucopyranosid
EDNRB	Endothelin receptor B
APC	Argan Press Cake
VC	Vitamin C
OPC	Oligomeric procyanidins
